# Phylogenetically Diverse *ure*C Genes and Their Expression Suggest the Urea Utilization by Bacterial Symbionts in Marine Sponge *Xestospongia testudinaria*

**DOI:** 10.1371/journal.pone.0064848

**Published:** 2013-05-31

**Authors:** Jing Su, Liling Jin, Qun Jiang, Wei Sun, Fengli Zhang, Zhiyong Li

**Affiliations:** Marine Biotechnology Laboratory, State Key Laboratory of Microbial Metabolism and School of Life Sciences and Biotechnology, Shanghai Jiao Tong University, Shanghai, P.R. China; Wageningen University, The Netherlands

## Abstract

Urea is one of the dominant organic nitrogenous compounds in the oligotrophic oceans. Compared to the knowledge of nitrogen transformation of nitrogen fixation, ammonia oxidization, nitrate and nitrite reduction mediated by sponge-associated microbes, our knowledge of urea utilization in sponges and the phylogenetic diversity of sponge-associated microbes with urea utilization potential is very limited. In this study, *Marinobacter litoralis* isolated from the marine sponge *Xestospongia testudinaria* and the slurry of *X. testudinaria* were found to have urease activity. Subsequently, phylogenetically diverse bacterial *ure*C genes were detected in the total genomic DNA and RNA of sponge *X. testudinaria*, *i.e.*, 19 operative taxonomic units (OTUs) in genomic DNA library and 8 OTUs in cDNA library at 90% stringency. Particularly, 6 OTUs were common to both the genomic DNA library and the cDNA library, which suggested that some *ure*C genes were expressed in this sponge. BLAST and phylogenetic analysis showed that most of the *ure*C sequences were similar with the urease alpha subunit of members from *Proteobacteria*, which were the predominant component in sponge *X. testudinaria*, and the remaining *ure*C sequences were related to those from *Magnetococcus*, *Cyanobacteria*, and *Actinobacteria*. This study is the first assessment of the role of sponge bacterial symbionts in the regenerated utilization of urea by the detection of transcriptional activity of *ure*C gene, as well as the phylogenetic diversity of *ure*C gene of sponge bacterial symbionts. The results suggested the urea utilization by bacterial symbionts in marine sponge *X. testudinaria*, extending our understanding of nitrogen cycling mediated by sponge-associated microbiota.

## Introduction

Nitrogen can be a limiting nutrient and nitrogen availability in the marine environment may therefore be a major factor in controlling biomass production. Most nitrogen cycling-related studies focus on nitrate and ammonium as the primary sources of nitrogen available to coral reefs [1], because they are generally the preferred forms for assimilation [2], [3]. But tropical marine ecosystems rely heavily on regenerated nitrogen sources [4]. In low nitrate systems, regenerated nitrogen such as urea or ammonium can provide up to 75% of the requirements of phytoplankton [5], [6]. Urea represents the single dominant component of the diverse group of organic nitrogenous compounds in the oligotrophic oceans [7]. Urea in the oceans originates from a variety of sources [8]–[12]. For example toadfish (*Opsanus beta*) can excrete over 90% of their waste nitrogen as urea [13]. On the other hand, urea can be used directly by many organisms such as hard corals [3], phytoplankton [5], benthic macroalgae [14], protozoans and bacteria [15], [16]. However, in total, the role of regenerated utilization of urea in the nitrogen cycle of marine ecosystem is poorly understood, especially for marine microbial symbionts.

Marine sponges (phylum Porifera), the oldest multicellular animals (metazoans), are one of the main components in coral reefs ecosystem. Sponges are filter feeders, pumping large amounts of seawater every day. Their ecological significance has attracted much research interest [17], [18], [19]. It is known that sponges host abundant and diverse symbiotic microorganisms including bacteria, archaea, unicellular algae and fungi [20]–[32]. In oligotrophic seawater where the nitrogen level is very low, symbiotic microorganisms may contribute to the nitrogen budget of sponges via fixation of atmospheric nitrogen [18]. The release of nitrate from incubated sponges provided the first indication of nitrification within these organisms, with estimated rates often far exceeding those for other benthic substrata [19]. To date, the potential of sponge microbial symbionts in nitrogen fixation, ammonia oxidization and nitrite reduction have been suggested by the analysis of nitrogen cycle-related functional genes such as *nif*H, *amo*A, *nir*K or *nir*S [21], . But, till now, the regenerated utilization of urea by sponge bacterial symbionts remains nearly unknown [38].

Urease is one of the important enzymes in nitrogen cycle [40]. Most organisms that use urea as a source of nitrogen rely on a urease such as urea amidohydrolase (EC 3.5.1.5), that can catalyze the hydrolysis of urea to yield ammonia and carbamate [41]−[44]. Bacterial urease is a trimer of three subunits (encoded by *ure*A, *ure*B, and *ure*C) and requires up to four accessory proteins for activation and Ni^+^ incorporation (most commonly encoded by *ure*D, *ure*E, *ure*F and *ure*G) [45]. The urease peptides have highly conserved active sites and Ni^+^ binding residues [44]. According to the single-cell genomics analysis of *Poribacteria* in sponge *Aplysina aerophoba*, a 10 ORF containing urease gene cluster including three urease subunits *ure*A, *ure*B, *ure*C was identified, suggesting that the *Poribacteria* are capable of using urea to cover their nitrogen needs [38]. However, we don’t know whether urease genes are active in sponges and what is the phylogenetic diversity of urease genes of microbial community in sponges.

The *ure*C gene was chosen as the target gene for urease analysis because it is the largest of the genes encoding urease functional subunits and contains several highly conserved regions that are suitable as PCR priming sites [46]. In this study, using *ure*C gene as marker, the phylogenetically diverse *ure*C genes and their expression in the marine sponge *X. testudinaria* were investigated for the first time, suggesting an important role of phylogenetically diverse sponge bacterial symbionts in the regenerated utilization of urea for sponge host.

## Methods

### Ethics Statement: N/A

This study and the collection of sponges were approved by the ethics committee for scientific study at Shanghai Jiao Tong University.

No legislation was required for the sampling of sponges around Yongxing island (112°20′E, 16°50′N). The government of China permits the sampling of sponge samples around the Yongxing Island in the South China Sea for scientific research, and no specific permissions were required for these locations/activities, the location is not privately-owned or protected in any way, the field studies did not involve endangered or protected species. The sponges were collected by us.

### Sample Collection

Specimens of the marine sponge *X. testudinaria* were collected by scuba diving at a depth of *ca.* 20 m from Yongxing Island (latitude 112°20′E, longitude 16°50′N) in the South China Sea, in 2010. Sponge samples were transferred directly to plastic bags to avoid the contact of sponge tissue with air and immediately stored at −80°C. Sponge samples for RNA extraction were immediately stored in RNA later (Qiagen, Valencia, CA, USA).

### Urease Activity Assay

Sponge samples were washed with sterile artificial seawater (1.1 g CaCl_2_, 10.2 g MgCl_2_·6H_2_O, 31.6 g NaCl, 0.75 g KCl, 1.0 g Na_2_SO_4_, 2.4 g Tris-HCl, 0.02 g NaHCO_3_, 1 L distilled water, pH 7.6) to remove the contaminants from sea water or sediment. Three sponge cubes (∼1 cm^2^) from different parts of the sponge were grounded into slurry using a mortar and pestle. The sponge slurry and bacterial isolates from the sponge were tested for urease activity using a colorimetric urea agar method at 28°C [47]. The medium contains 1 g peptone, 1 g glucose, 2 g KH_2_PO_4_, 0.012 g phenolsulfonphthalein, 20 g agar, 1L artificial seawater (ASW). The medium was supplemented with 40% (w/v) urea solution [47]. Cultures were scored positive for urease activity if the agar color changed from pale orange (pH 6.9) to pink or fuchsia (pH 7.6 or higher). The color change is because of ammonia formation resulting from ureolysis [46]. The uninoculated control should be still blank.

### Total Genomic DNA and RNA Extraction

Three cubes (∼1cm^2^) from different parts of the sponge were dissociated in sterilized CMFSW (31.6 g NaCl, 0.75 g KCl, 1.0 g Na_2_SO_4_, 2.4 g Tris-HCl, 0.02 g NaHCO_3_, 7.2 g EDTA, 1L distilled water, pH 7.6) and grinded into slurry using a mortar and pestle. Sponge slurry was centrifuged at 100×g for 15 min to separate and discard the skeletal components. The supernatants were freeze-dried and grinded into powder, which was washed twice with 1 ml TE and centrifuged at 12,000×g for 1 min. Total DNA and RNA were extracted by AllPrep DNA/RNA Mini Kit (Qiagen, Germany) simultaneously; RNA extraction were treated with amplification grade DNase I (Invitrogen) according to the manufacturer’s protocol to remove any residual DNA prior to reverse transcription for cDNA synthesis. The amount and purity of DNA or RNA were determined by NanoDrop Spectrophotometer ND-1000 (Thermo Fisher Scientific, Wilmington, USA), based on the 260 nm absorption and the 260/280 ratio, respectively.

### Reverse Transcription and cDNA Synthesis

RNA was reverse-transcribed into cDNA using the SuperScript III First Strand Synthesis System (Invitrogen). Reverse transcription was carried out with random hexamer primers in 25 µl reaction system containing 100 ng template RNA, 500 nmol of reverse primer, 1.5 µl dNTPs, H_2_O was added to reach 12.5 μl. After incubation at 65°C for 5 min, the 25 µl reaction mixture was placed on ice for 1 min, added 12.5 µl cDNA Synthesis Mix (10×RT Buffer 2.5 µl, 25 mM MgCl_2_ 5 µl, 0.1 M DTT 2.5 µl, RNaseOUT 1.25 µl, SuperScript III RT 1.25 µl), incubated at 50°C for 50 min and the reaction was terminated at 85°C for 5 min [48]. The cDNA obtained was stored at −20°C until further use.

### PCR Amplification of Urease (*ure*C) Genes from Genomic DNA and cDNA

Bacterial urease (*ure*C) genes were amplified with the primer set L_2_F (5′-ATHGGYAARGCNGGN AAYCC-3′) and L_2_R (5′-GTBSHNCCCCARTCYTCRTG-3′) [46] from genomic DNA and cDNA. Reactions were performed in Mastercycler personal (Eppendorf). The PCR mixture (20 µl) contained 100 ng template DNA, 500 nmol of each primer, 10 µl 2×PCR Mix, 0.5 µl DNA polymerase and H_2_O was added to reach 20 µl. PCR amplification began with a 5 min denaturing step at 94°C, followed by 30 cycles at 94°C for 1 min, 57°C for 1.5 min, and 72°C for 2 min. Extension was achieved at 72°C for 10 min. PCR products (390 bp) were purified by electrophoresis on a 2% (wt/vol) agarose gel and recovered using a gel purification kit (Takara, Dalian, China).

### PCR Amplification of 16S rRNA Gene from Genomic DNA

Bacterial 16S rRNA genes were amplified using the domain bacteria-specific primer 27F (5′-GAGTTTGATCCTGGCTCAG-3′) and universal primer 1500R (5′-AGAAAGGAGGTGATCCA GCC-3′) [49]. PCR Kit (Takara, Dalian, China) was used in the PCR amplification. The PCR mixture (20 µl) contained 100 ng template DNA, 500 nM of each primer, 10 µl 2×PCR Mix, 0.5 µl DNA polymerase and H_2_O was added to reach 20 µl. Cycling conditions were as follows: initial denaturation at 94°C for 3 min, 30 cycles of 94°C for 1 min, 54°C for 1 min, and 72°C for 2 min, and a final extension of 10 min at 72°C [50]. PCR products of 1500 bp were purified by electrophoresis on a 1% (wt/vol) agarose gel and recovered using a gel purification kit (Takara, Dalian, China).

### Clone Library Construction and Sequencing

The libraries of *ure*C (from genomic DNA and cDNA) and 16S rRNA genes were separately constructed using the pMD18-T Cloning kit (Takara, Dalian, China) following the manufacturer’s instructions. Transformants were inoculated into LB broth (with 100 µg/ml ampicillin) and incubated overnight at 37°C. The positive recombinants were screened on X-Gal (5-bromo-4-chloro-3-indoly-b-D-Galactopyrano-side) -IPTG (isopropyl- b-D- thiogalactopyranoside)-ampicillin-tetracycline indicator plates by color-based recombinant selection. Positive clones were identified by PCR amplification with pMD18-T vector primer pairs: M13F (5′-TGTAAAACGACGGCCAGT-3′) and M13R (5′-CAGGAAACAGCTATGACC-3′), using the same program as the PCR amplification of *ure*C gene or 16S rRNA gene.

### Species Richness Estimation and Phylogenetic Analysis

Operational taxonomic unit (OTU) was defined at the 97% similarity (*3*% difference) for 16S rRNA gene and 90% similarity (10% difference) for *ure*C gene, respectively. Chimera checking was carried out using the program Bellerophon v3.0 from the Greengenes website to check the bacterial 16S rRNA genes [51]. Meanwhile Abundance-based coverage estimator (ACE), Chao1, Shannon, Simpson and rarefaction analysis were performed using DOTUR [52]. One representative clone was selected from each OTU for further phylogenetic analysis. The nearest relatives of each sequence were obtained from the GenBank database using the blastn tool (http://blast.ncbi.nlm.nih.gov/). The 16S rRNA gene sequence was used for the BLAST analysis of bacterial diversity and the deduced amino acid sequence was used for the BLAST analysis of *ure*C gene. The unrooted phylogenetic tree was constructed using Clustal X 2.0 and Mega 5.0. At the same time, all 16S rRNA gene sequences were classified using the Ribosomal Database Project (RDP) (http://rdp.cme.msu.edu/index.jsp) classifier with a confidence threshold of 70%.

### Nucleotide Sequence Accession Numbers

The gene sequences were deposited in the GenBank database under the accession numbers: JQ359624-JQ359642 (*ure*C gene in genomic DNA library), KC152840-KC152847 (*ure*C gene in cDNA library), JQ359612-JQ359623 (16S rRNA gene library), KC763777 (16S rRNA gene of *Marinobacter litoralis*).

## Results

### The Urea Utilization by Sponge *X. testudinaria*

**Figure 1:**
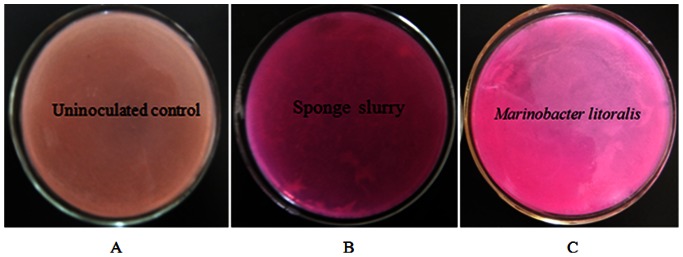
Urease activity of marine sponge *X.* testudinaria. A: negative control; B: activity of sponge slurry; C: *Marinobacter litoralis* (bacterium isolated from the sponge).

In the urea utilization test of sponge *X. testudinaria*, the color of the agar plate which was coated with sponge slurry changed from yellowish to pink within several hours (Figure 1A), the color of the negative control didn’t change (Figure 1B). Apparently, the homogenates of the sponge contained urease which might be produced by sponge itself or its associated microorganisms. In the former study, a bacterium *Marinobacter litoralis*, which was isolated from the sponge *X. testudinaria*, showed similar color change in the agar plate (Figure 1C). *M. litoralis* was also tested to be urease-positive by Yoon et al. [53]. Thus, it is suggested that sponge-associated bacteria may participate in the urea hydrolysis. In order to reveal the diversity of *ure*C gene of the bacterial symbionts in *X. testudinaria*, the bacterial *ure*C gene was analyzed.

### The Detection of *ure*C Gene and its Expression in Sponge *X. testudinaria*

**Figure 2:**
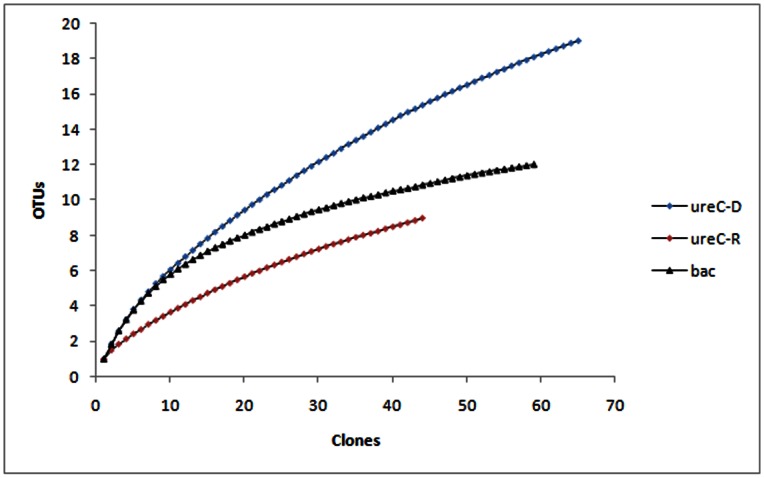
Rarefaction curves of *ure*C and 16S rRNA gene sequences. (Clusterization stringency at 90% and 97% for *ure*C and 16S rRNA gene, respectively.).

After the *ure*C gene was successfully amplified from the total genomic DNA and cDNA, two clone libraries of *ure*C genes were constructed, respectively named *ure*C-D library (genomic DNA library) and *ure*C-R library (cDNA library). The coverage ratios of *ure*C-D and *ure*C-R libraries were 84.61% and 88.16%, respectively (Figure 2), suggesting that the sequencing of clones was nearly saturated.

**Figure 3:**
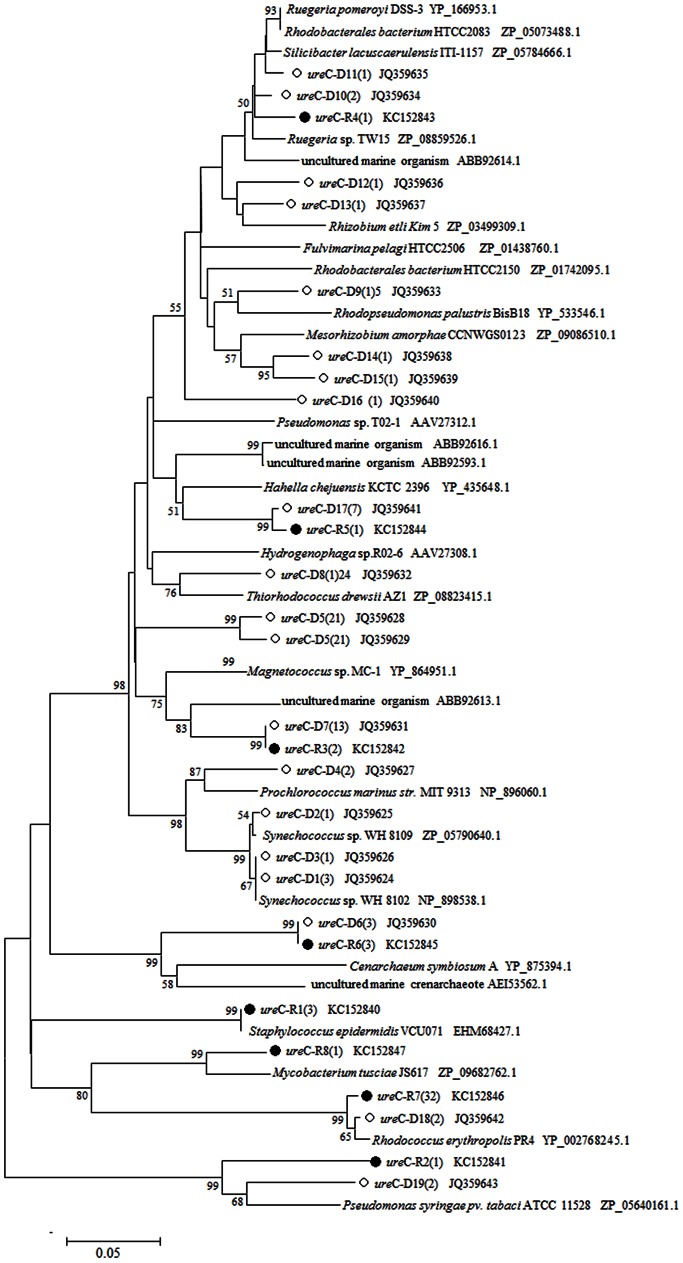
Unrooted phylogenetic tree based on urease alpha subunit (130aa) of sponge *X.* *testudinaria* using Neighbour-joining method. The *scale bar* represents 0.05 substitutions per amino acids position. Bootstrap values (1,000 replicates) higher than 50% are shown. ○mark and *ure*C-D mean the OTU in genomic DNA library, and •mark and *ure*C-R mean the OTU in cDNA library. The number inside the parenthesis means the number of sequences within each OTU.

A total of sixty five positive clones were randomly selected from the *ure*C-D library for sequencing. Finally, 19 OTUs were obtained at the 10% dissimilarity level. BLAST analysis using deduced amino acid sequence and phylogenetic analysis showed that the most similar reference sequences were from *Proteobacteria*, *Cyanobacteria*, and *Actinobacteria* (Figure 3).

In the *ure*C-R library, a total of forty five positive clones were randomly selected for sequencing, resulting in 8 OTUs. In particular, six OTUs were common to both the genomic DNA library and the cDNA library: *ure*C-D6(3) and *ure*C-R6(3), *ure*C-D7(13) and *ure*C-R3(2), *ure*C-D10(2) and *ure*C-R4(1), *ure*C-D17(7) and *ure*C-R5(1), *ure*C-D18(2) and *ure*C-R7(32), *ure*C-D19(2) and *ure*C-R2(1)). Therefore, RT-PCR results showed that some of the sponge-associated *ure*C genes were metabolically active. BLAST analysis showed that these active *ure*C genes were of diverse microbial origin, suggesting various groups of sponge-associated microbes might participate in the urea transformation for sponge host. The finding of phylogenetically diverse bacterial urease genes and their expression strongly suggested the bacterial role in the urea utilization of sponge *X. testudinaria*.

### Phylogenetic Diversity of Bacteria in Sponges

**Figure 4:**
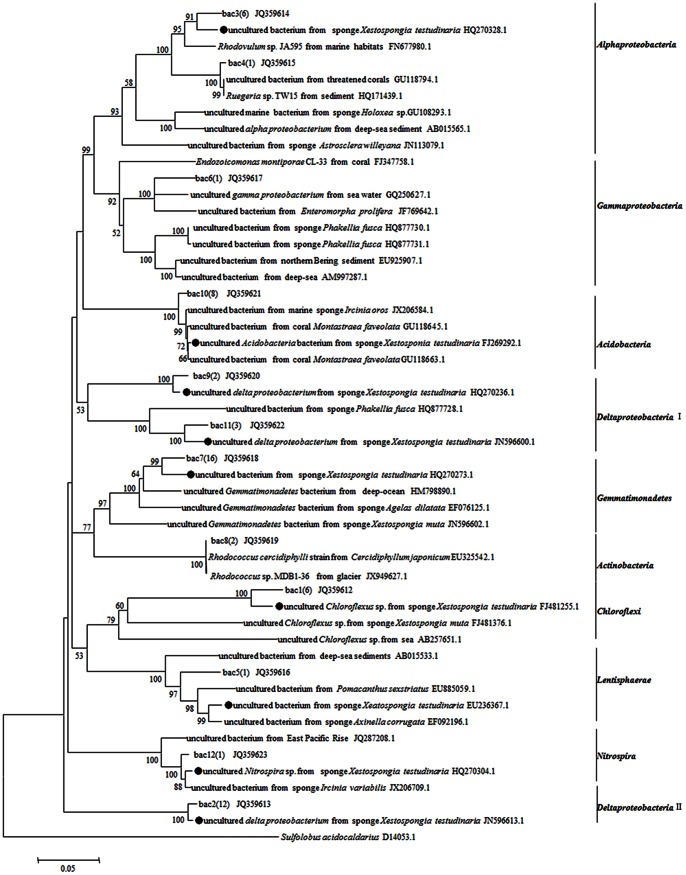
Unrooted phylogenetic tree based on bacterial 16S rRNA gene sequences (*ca*.1,400bp) of sponge *X.* *testudinaria* using Neighbour-joining method. The *scale bar* represents 0.05 substitutions per nucleotide position. Bootstrap values (1,000 replicates) higher than 50% are shown. The number in brackets shows the number of sequences in each OTU. •mark means sequences from sponge *X. testudinaria.*

**Figure 5:**
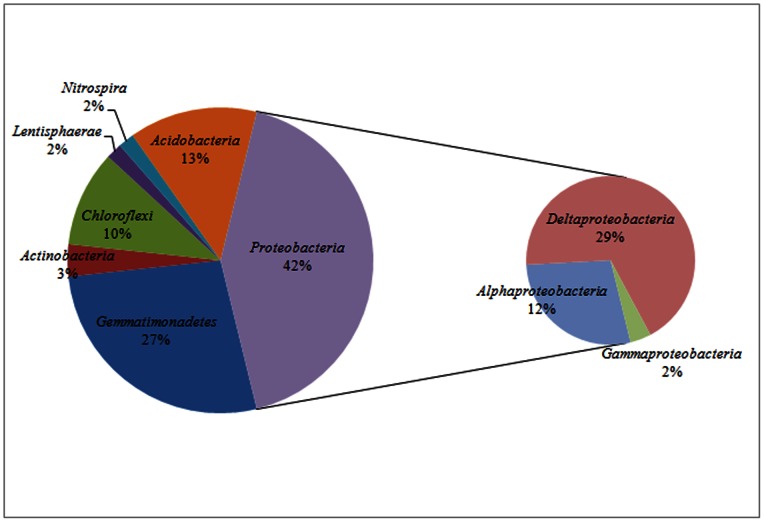
Pie charts illustrating the bacterial community based on 16S rRNA gene libraries of sponge *X.* *testudinaria*. Sequences in libraries were classified using the Classifier Tool provided by the Ribosomal Database Project. Hierarchical taxa assignment was based on RDP naive Bayesian rRNA Classifier. Percentage represented specific value between the number of clones in each taxon and the number of all clones in 16S rRNA gene library.

Fifty nine clones from the16S rRNA gene library were sequenced successfully. At the 3% dissimilarity level, these sequences could be divided into 12 OTUs. Rarefaction analysis indicated that the bacterial library well represented the microbial communities because the rarefaction curve was approaching plateaus. The Chao1 and ACE richness estimators predicted 16.03 and 12.58 OTUs at the species level for sponge *X. testudinaria*. The Shannon and 1/Simpson diversity indicated at the species level were 2.07 and 6.87. BLAST analysis of 16S rRNA gene indicated that, 9/12 bacterial representatives, except for clones bac4(1), bac6(1) and bac8(2), were closely related to the uncultured relatives from marine sponge *X. testudinaria* in the Pacific Ocean [54] (Figure 4), which indicated that *X. testudinaria*-derived 16S rRNA gene sequences were more closely related to each other than to 16S rRNA gene sequences derived from other samples. Meanwhile, nearly one half sequences had a very low similarity (≤94%) with related sequences in GenBank indicating the novelty of bacteria in the sponge *X. testudinaria* from the South China Sea. All bacterial sequences were classified using the RDP classifier with a confidence threshold of 70%. The most predominant bacterial phylum observed in sponge *X. testudinaria* was *Proteobacteria*, including *Alphaproteobacteria* (28%), *Deltaproteobacteria* (68%), and *Gammaproteobacteria* (4%). The second abundant phylum was *Gemmatimonadetes* (27%). The remaining belonged to *Acidobacteria* (14%), *Actinobacteria* (3%), *Chloroflexi* (10%), *Nitrospira* (2%) and *Lentisphaerae* (2%) (Figure 5).

## Discussion

Marine sponge *X. testudinaria* and *M. litoralis* which was isolated from the sponge can transfer urea into ammonia, which suggests the potential for urea utilization by sponge symbionts. This suggestion was supported by the detection of bacterial *ure*C gene and its expression in this sponge. Among the observed 19 OTUs of *ure*C genes observed in the *ure*C-D library, only 6 OTUs were detected in the *ure*C-R library, suggesting that not all the *ure*C genes were active. In contrast, two OTUs, *ure*C-R1 which was closely related to *Staphylococcus epidermidis* VCU071, and *ure*C-R8 which was closely related to *Mycobacterium tusciae* JS617, were unique in cDNA library. It was probably due to the incomplete sequencing of clones in the two libraries. Unexpectedly, in *ure*C-D library, we also acquired one *ure*C gene which was most similar to *Cenarchaeum symbiosum* A. Previous study showed that *Thaumarchaeota* from arctic deep waters had high abundance of urease genes [55]. This finding indicated that archaea might also play an important part in urea utilization.

Montalvo et al. [54] analyzed the bacterial diversity of *X. testudinaria* from the Manado Bay using gene library-based method, and found that *Chloroflexi*, *Acidobacteria*, *Actinobacteria* and *Deltaproteobacteria* were dominant in the bacterial community. Using 454 pyrosequencing, Lee et al. [30] revealed that *Proteobacteria*, *Firmicutes* and *Chloroflexi* were predominant bacterial phyla in *X. testudinaria* from the Red Sea. In this study, the bacterial community in *X. testudinaria* from the South China Sea was found to be composed of *Proteobacteria* (42%), *Gemmatimonadetes* (27%), *Acidobacteria* (14%), *Chloroflexi* (10%), *Actinobacteria* (3%), *Nitrospira* (2%) and *Lentisphaerae* (2%). As Montalvo et al. [54], dominant *Deltaproteobacteria* (68%), *Chloroflexi*, *Acidobacteria* and *Actinobacteria* were observed in *X. testudinaria*, and meanwhile as Lee et al. [30], *Proteobacteria* and *Chloroflexi* were detected. Previous studies concluded that uniform microbial community occurred in sponges from different oceans [56]−[59]. According to the comparison above, though the sponge *X. testudinaria* species were collected from different ocean areas, it had similar predominant bacterial groups, *i.e. Proteobacteria*, *Chloroflexi*, *Acidobacteria* and *Actinobacteria* might represent its core bacterial community. This is supported by Figure 4, where *X. testudinaria*-derived 16S rRNA gene sequences were clustered together suggesting some bacteria are *X. testudinaria* specific. Besides, this study, together with that of Lee et al. [30], Montalvo et al. [54], and Schmitt et al. [31], suggested the sponge species-specific bacterial components which might come from the different marine environment, since sponge microbial symbionts might be acquired by both horizontal transfer and vertical transmission approaches [26], [28].

The phylogenetic tree based on *ure*C genes indicated that *Proteobacteria*, *Cyanobacteria*, *Actinobacteria* and *Cenarchaeum* might produce urease in the sponge *X. testudinaria*. However, according to the phylogenetic analysis of 16S rRNA genes, the dominant bacterial phyla existing in the sponge *X. testudinaria* were *Proteobacteria*, *Gemmatimonadetes*, *Acidobacteria* and *Chloroflexi*. Apparently, 16S rRNA-based phylogeny showed partial congruence to the *ure*C-based phylogeny. Probably, the discrepancy may be due to events of horizontal transfer of *ure*C among ureolytic bacterial species. Investigating the 16S rRNA gene can reveal comprehensive community structure of sponge-associated bacteria, but is not a good approach for ecological investigation of ureolytic bacterial species, while the *ure*C gene can provide a better estimation of bacteria with urea utilization potential. So, integrated approach combining the 16S rRNA gene (phylogenetic marker) and *ure*C gene (functional marker), should be more accurate in the analysis of ureolytic bacteria.

Most of the marine sponges live in coral reefs ecological system, where it is generally limited by nitrogen. The nickel metalloenzyme urease catalyses the hydrolysis of urea to ammonia and carbamate, and thus generates the preferred nitrogen source of many organisms. Urease can be produced by diverse bacterial species [60]. Urea in sponges may come from the sponge host excretion, seawater, bacterial degradation of nucleic and amino acids, and is therefore a possible product to be encountered in the sponge mesohyl. Only the potential of sponge-associated bacteria in urea utilization was suggested by genome analysis before this study [38]. In this study, the plate assay and phylogenetic analysis suggested the function of sponge bacterial symbionts in urea utilization. This study provided the first insight into the bacterial potential in urea utilization by detecting the transcriptional activity of *ure*C gene as well as the phylogentic diversity of bacteria with *ure*C gene.
